# Mapping social determinants of cognitive health in Canada: a scoping review

**DOI:** 10.24095/hpcdp.45.11/12.01

**Published:** 2025

**Authors:** Sarah O’Connor, Teodora Riglea, Mathilde Lavigne-Robichaud, Lucie Lapierre

**Affiliations:** Public Health Capacity and Knowledge Mobilization Unit, Operation and Emergency Management Branch, Public Health Agency of Canada, Montral, Quebec, Canada

**Keywords:** dementia, social determinants of health, scoping review, Alzheimer disease, cognition, education, social support

## Abstract

**Introduction:**

We sought to map the literature assessing the associations between the social determinants of health (SDoH) and the cognitive health of adults in Canada.

**Methods:**

We searched the Embase, CENTRAL, Global Health and MEDLINE databases through Ovid, from inception to 20 February 2024, for studies examining associations between SDoH and cognitive health among Canadian adults, irrespective of health or cognitive status.

**Results:**

We identified 159 publications covering 93 studies; 27% (n = 25) had nationwide coverage and 48% (n = 45) were from Ontario or Quebec. Of the 410 associations between SDoH and cognition, 20 were from 6 qualitative studies and 390 from 87 quantitative studies. Education was the most frequently evaluated (46%) of the 29 identified SDoH categories, then social support (24%), household/individual income(19%), marital status (17%), occupation (16%), rural or urban area of residence (16%), living arrangement/household composition (12%) and environmental factors (13%). Two-thirds (67%) of the studies examined various cognitive health constructs, while 41% evaluated dementia (all types). Most of the SDoH were from the settings with which individuals directly interact. SDoH related to environmental exposure or pollution, societal norms, beliefs, values and practices were less frequently evaluated.

**Conclusion:**

This scoping review provides a detailed map of the literature on SDoH and cognitive health in Canada. It highlights the importance of considering a comprehensive range of SDoH and of using diverse data sources and data collection approaches. The results also highlight SDoH that remain largely unexamined and should be prioritized in future research.

**Highlights**

- The 93 studies we reviewed identified 29 categories of social determinants of health (SDoH) associated with the cognitive health of adults in Canada.
- Education and social support were most frequently examined SDoH.
- Most of the examined SDoH were within people’s immediate environments, while systemic and structural SDoH were less frequently examined.
- Almost half of the studies were conducted in Ontario or Quebec, and there were no data specific to Yukon, the Northwest Territories, Nunavut, Nova Scotia or Prince Edward Island.
- Only two interventional studies evaluated the impact of SDoH on cognitive health, which highlights a gap in the integration of health impact assessments in public health initiatives.

## Introduction

Between 2022 and 2023, approximately 487 000 (6.2%) of Canadians aged 65 years and older were living with diagnosed dementia, with 99 000 newly diagnosed during this period.^1^ While the incidence rate of dementia in this age group has been decreasing over the past decade, the overall number of Canadians with dementia is expected to increase because of the aging population.^2^ In response to the urgent need to understand and prevent dementia, the Parliament of Canada passed the *National Strategy for Alzheimer’s Disease and Other Dementias Act*^3^ in 2017. In 2019, the federal Minister of Health released the first national strategy, *A Dementia Strategy for Canada: Together We Aspire*,^4^ and subsequently, related annual reports.^5^,^6^

In 2024, the *Lancet* Commission on dementia estimated that 45.3% of dementia cases could be explained by 14 risk factors: 1)less education; 2)hearing loss; 3)high LDL cholesterol; 4)depression; 5)traumatic brain injury; 6)physical inactivity; 7)diabetes; 8)smoking; 9)hypertension; 10)obesity; 11)excessive alcohol use; 12)social isolation; 13)air pollution; and 14)vision loss.^7^ While many of these risk factors can be modified at the individual level, not everyone has access to the resources necessary to address them effectively.^8^,^9^ As the Alzheimer Society of Canada noted, “These risk factors are only truly modifiable if the proper supports are provided by our communities, public health agencies and other governmental organizations.”^8^^,p.50^

Social determinants of health (SDoH)—the conditions in the environments where “people are born, grow, live, work and age”^10^,^p.76^—profoundly influence people’s exposure to or ability to modify the risk factors identified by the *Lancet* Commission.^7^,^8^ The differences in the distribution of SDoH across subgroups and populations derive from established systemic social, political and economic structures, which lead to socioeconomic and health inequalities and, ultimately, disparities in health outcomes, including cognitive diseases.^11^

The framework proposed by Adkins-Jackson et al.^11^ incorporates Bronfenbrenner’s ecological theory^12^ to illustrate how SDoH influence the risk of dementias classified as Alzheimer disease. This framework depicts a continuum of embedded and interdependent systems: the microsystem (the settings with which individuals directly interact); the mesosystem (interactions between microsystems); the exosystem (the factors that indirectly influence health, such as climate change); the macrosystem (societal norms, beliefs, values and practices established in the society in which individuals live that influence microsystems); and the chronosystem (exposure to SDoH during the life course)^11^ (see Supplemental Figure 1 at https://osf.io/w4mqc). As such, key SDoH that influence cognitive health and dementia include the 14 risk factors identified by the *Lancet* Commission^7^ as well as neighbourhood deprivation, workplace conditions and occupation, financial and food insecurity, poverty, housing and other factors. These, in turn, are influenced by broader systemic SDoH such as structural racism, discrimination or ageism.^13^

**Figure 1:**
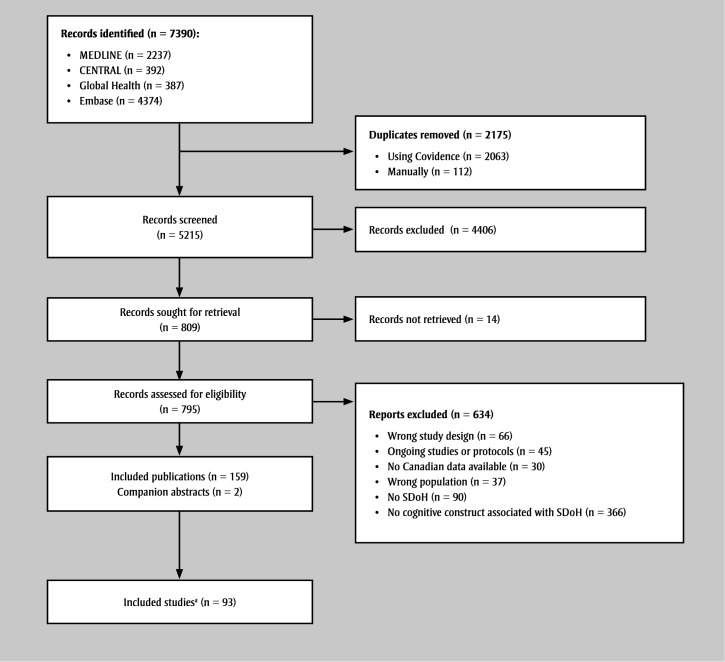
PRISMA-ScR flow diagram of included studies

**Abbreviations: **PRISMA-ScR, Preferred Reporting Items for Systematic Reviews and Meta-analyses extension for Scoping Reviews; SDoH, social determinants of health.

^a^ One publication had data from two distinct sources.

Although Canada’s national dementia strategy^3^ includes prevention as a core objective, it primarily focuses on the individual risk factors identified by the *Lancet* Commission;^7^ targeting the broader structural determinants of these risk factors is less evident. Given that the interdependence of the SDoH systems can result in a cascade of effects on individuals’ capacity to act upon modifiable risk factors and take action to prevent diseases,^11^ it is essential to consider the structural SDoH that shape health disparities and the incidence of cognitive diseases in Canada. Although interest in the role of SDoH in cognitive health and dementia is increasing,^8^ we do not yet have a comprehensive synthesis of the existing Canadian scientific literature examining associations between SDoH and cognition.

The objective of this scoping review was to map the current state of knowledge on the SDoH that affect cognitive health among adults in Canada.

## Methods

This scoping review was conducted according to JBI’s guide for scoping reviews^14^ and the six-stage approach from Arksey and O’Malley^15^ and Levac et al.^16^ We followed the Preferred Reporting Items for Systematic Reviews and Meta-Analyses extension for Scoping Reviews (PRISMA-ScR).^17^ The protocol is available at https://osf.io/764ks.

***Eligibility criteria***

We included studies that assessed all levels of cognitive health status (i.e. healthy cognitive status, mild cognitive impairment or diagnosed dementia [all types]) in samples of adult participants aged 18 years or older or in samples of youth and adults composed mainly (≥80%) of adults. No restrictions were imposed on pre-existing health conditions, including diseases that may affect cognitive health. Although we did not include studies that focused on caregivers as the unit of analysis, we included studies in which the presence of caregivers was considered an SDoH for cognitive health. 

Only those studies examining populations living in Canada were included. Multicountry studies were included if we could extract results specific to Canada.

We included studies if they evaluated an association between SDoH and at least one aspect of cognitive health. We initially limited SDoH to the World Health Organization definition^10^ but subsequently expanded our examination to include those identified in the Adkins-Jackson et al. framework^11^ and others that may be related to cognitive health.^18^

We excluded studies that assessed individual nonmodifiable risk factors (e.g. age, sex, genotype) or modifiable risk factors (e.g. diet, physical activity, tobacco use, clinical measurements [anthropometric measurements, blood pressure, lipid profile, glucose profile]) without evaluating SDoH. Macrosystem-level risk factors (e.g. gender) were considered to be SDoH if specific or indirect assessments of societal norms, beliefs, values, practices or points of view were conducted (e.g. genderism—the belief that there are only two genders and that a person’s gender should align with their sex as assigned at birth).

Cognitive health is a global concept that includes (but is not limited to) dementia and the overall progression towards dementia. We defined cognitive health broadly as the ability to think clearly, to learn and to remember.^19^ (Definitions for the different cognitive health constructs considered in this scoping review are listed in Supplemental Table 1 at https://osf.io/w4mqc.) The tools used to evaluate cognitive constructs included self-reported outcomes, standardized or home tests, questionnaires, medical diagnosis screenings and cognitive evaluations (see Supplemental Table 2 at https://osf.io/w4mqc).

The following constructs were not considered because they are only indirectly associated with cognitive health: mental health or psychiatric conditions (e.g. depression, anxiety, psychosis, schizophrenia or bipolar disorder, attention deficit disorder with or without hyperactivity), intelligence tests, and motor impairments or related diseases (e.g. Parkinson disease, multiple sclerosis). 

We included quantitative and qualitative peer-reviewed studies as well as conference abstracts. In quantitative studies, only univariate and multivariate associations where SDoH were considered the main exposure or a predictor were included; SDoH used as adjustment variables were not. Qualitative studies of perceived changes in cognitive health (or symptoms of) in the presence of a specific SDoH were included. We included both empirical and conceptual studies as long as they reported an association between SDoH and cognition. Reviews, government policy documents and protocols were excluded. 

There were no restrictions on the year of publication, although conference abstracts were limited to the last 5 years (2019–2024).

***Search and study selection***

We searched the Cochrane Central Register of Controlled Trials (CENTRAL), Embase, Global Health and MEDLINE biomedical databases through Ovid, from their inception until 20 February 2024, using both indexed and free keywords associated with SDoH, cognitive health and Canadian contexts (see Supplemental Table 3 at https://osf.io/w4mqc). The search strategy was reviewed and validated by a science librarian. 

Citations were imported into Covidence (Veritas Health Innovation, Melbourne, AU) and duplicates were removed. Two reviewers [TR, SO] independently screened the titles, abstracts and full texts of the retrieved studies. Any differences were resolved by other reviewers [MLR, LL]. The search strategy was re-evaluated iteratively. Because of the large number of eligible studies, we restricted the search to biomedical databases only.

***Data extraction and charting***

Data were extracted by one reviewer [TR or SO] into a pre-piloted data collection form. The following details were collected from each retrieved publication: year of publication, study design, name of the study, sample size and examined SDoH, cognitive health constructs and their associations. As the assessment of the effects of exposures was not an objective, we did not appraise risk of bias.

To avoid overestimating the frequency of associations, we combined articles that used the same cohort and treated them as one study. 

Using each study as the unit of analysis, we gathered information on the study participants’ age, sex, rural versus urban area of residence, health conditions, cognitive status at baseline and location in Canada. Identical associations in a single study were combined. In each study, we identified all the associations between individual cognitive health constructs and each given SDoH. Thus, if a study reported on several associations between a specific SDoH and different cognitive health constructs, each association was counted independently of the others. In the case of longitudinal studies with multiple time points, we selected the most recent time point. Measures assessing similar SDoH were combined into the same categories and classified according to the Adkins-Jackson et al. framework.^11^

When SDoH were assessed using indirect measures or proxies, we categorized them with the closest to the SDoH of interest. For example, self-reported race or ethnicity would be categorized in the macrosystem as an indirect measure of potential racism. 

We summarized data on SDoH, cognitive health constructs and their associations using frequencies or proportions. Associations reported in qualitative studies are summarized separately.

## Results

***Search results***

Of the 7390 records retrieved, 159 publications were included in this scoping review (see Figure 1). Of these, 158 were in English and 18 had abstracts in French. Articles were published between 1991 and 2024, with 101 published since 2014 and 72 since 2019. The sample sizes for both the qualitative and quantitative studies ranged from 3 to 6538000 participants. Of the included publications, 85 had cross-sectional designs, 49 retrospective or prospective cohort designs, eight case-control or nested case-control designs, six qualitative or mixed designs, six repeated cross-sectional designs, one case-crossover design, one randomized controlled trial, one pre–post design with no control group and one post hoc analysis of a nonrandomized controlled trial. (See Supplemental Table 4 at https://osf.io/w4mqc for the details of included publications.)

***Study characteristics***

The 159 retrieved publications covered 93individual studies. Of these, 25 had national coverage or covered multiple provinces, and almost half (n = 45) were conducted in Ontario or Quebec. (See Table 1 for summaries of study participants’ characteristics and the contexts of the retrieved studies; for additional details, see Supplemental Table 5 and 6 at https://osf.io/w4mqc.)

**Table 1:** Details of studies (n = 93) in the publications included in this scoping review

Characteristics	n (%)^a^
Location	
Multiple provinces or nationwide coverage	25 (27)
British Columbia	4 (4)
Alberta	7 (8)
Saskatchewan	4 (4)
Manitoba	2 (2)
Ontario	25 (27)
Quebec	20 (22)
New Brunswick	1 (1)
Nova Scotia	0
Prince Edward Island	0
Newfoundland and Labrador	2 (2)
Yukon, Northwest Territories and Nunavut	0
Not reported	3 (3)
Sex of participants	
Both	72 (77)
Men	6 (7)
Women	4 (4)
Not reported	11 (12)
Age group	
Young adults	1 (1)
Older adults	36 (39)
Adults (no specification)	56 (60)
Main type of data used	
Administrative database	21 (23)
Clinical data	8 (9)
Medical records	4 (4)
Survey data	29 (31)
Qualitative data	6 (6)
Mixed	25 (27)
Study population	
Ambulatory clinics	17 (18)
Community dwellings	59 (63)
First Nations, Inuit or Mtis communities	3 (3)
Emergency departments	1 (1)
Home care settings	2 (2)
Hospital settings	4 (4)
Long-term care facilities	6 (6)
Correctional facilities	1 (1)
Cognitive status at baseline	
Cognitively healthy	17 (18)
Cognitive impairment or dementia	6 (6)
Mixed	30 (32)
Not reported	40 (43)

^a^ Percentages are based on the total number of studies retrieved (n = 93).

We sorted the identified SDoH into 29 categories (see Table 2). A total of 80 studies (86%) assessed microsystem-level SDoH, for example, education (46%), social support (24%), household/individual income (19%), occupation (16%), urban or rural area of residence (16%), marital status (17%) and living arrangement/household composition (12%). Twelve studies (13%) evaluated exposure to environmental pollutants, an exosystem-level SDoH, and 31 (33%) assessed macrosystem-level SDoH, including access to health care (11%), ethnicity (11%), primary language or language barriers (11%) and First Nations, Inuit or Mtis identity (4%). Some SDoH were evaluated retrospectively over the lifespan (the chronosystem), the most common being highest educational attainment and past occupational exposure to pollutants. One study assessed the history of childhood maltreatment, and one evaluated perinatal and childhood exposure to mercury. None of the studies retrieved assessed interactions between different microsystems (i.e. the mesosystem).

**Table 2:** Frequency and proportions of studies (n = 93) examining each SDoH in this scoping review

SDoH^a^	n (%)		
	All studies (n = 93)	Quantitative studies (n = 87)	Qualitative studies (n = 6)
Microsystem^b^			
Education	43 (46)	42 (48)	1 (17)
Social support	22 (24)	18 (21)	4 (66)
Income	18 (19)	18 (21)	0
Marital status	16 (17)	16 (18)	0
Occupation or employment	15 (16)	15 (17)	0
Area of residence (rural/urban)	15 (16)	13 (15)	2 (33)
Immigration status	11 (12)	10 (11)	1 (17)
Living arrangement/household composition	11 (12)	11 (13)	0
Built environment	10 (11)	10 (11)	1 (17)
Housing	10 (11)	9 (10)	0
Neighbourhood deprivation or socioeconomic status	8 (9)	8 (9)	0
Childhood maltreatment	6 (6)	5 (6)	1 (17)
Occupational exposure	4 (4)	4 (5)	0
Loneliness	4 (4)	4 (5)	0
Food security	1 (1)	1 (1)	0
Exosystem^c^			
Environmental pollutants	12 (13)	12 (14)	0
Macrosystem^d^			
Access to health care	10 (11)	8 (9)	2 (33)
Ethnicity	10 (11)	10 (11)	0
Primary language or language barriers	10 (11)	9 (10)	1 (17)
First Nations, Inuit or Mtis identity	4 (4)	3 (3)	1 (17)
Knowledge of cognitive health	2 (2)	1 (1)	1 (17)
Policy implementation	2 (2)	2 (2)	0
Religion	2 (2)	1 (1)	1 (17)
Sexual orientation	2 (2)	2 (2)	0
Stigma	2 (2)	1 (1)	1 (17)
Culture	1 (1)	0	1 (17)
Gender identity or genderism	1 (1)	1 (1)	0
Marginalization	1 (1)	1 (1)	0
Quality of care	1 (1)	0	1 (17)

**Abbreviation: **SDoH, social determinants of health. 

^a^ Measures assessing similar SDoH were combined into the same categories and classified according to the Adkins-Jackson et al. framework.11 When SDoH were assessed using indirect measures or proxies, we categorized them with the closest SDoH of interest. 

^b^ The microsystem describes the settings with which individuals directly interact. 

^c^ The exosystem describes the factors that indirectly influence health. 

^d^ The macrosystem describes the societal norms, beliefs, values and practices. 

A total of 62 studies assessed either global cognition using, for example, the Mini Mental State Examination, the Montreal Cognitive Assessment (MoCA), the Cognitive Performance Scale, or global cognition composite measures, or a specific cognitive health construct using, for example, tests for executive function, processing speed or memory. A diagnosis of dementia (all types) was an outcome associated with SDoH in 38 studies (see Table 3; for additional information on the SDoH and cognitive constructs evaluated across studies, see Supplemental Table 5 at https://osf.io/w4mqc).

**Table 3:** Distribution of cognitive health constructs across quantitative and qualitative studies (n = 93) in this scoping review

Cognitive health constructs	n (%)		
	All studies (n = 93)	Quantitative studies (n = 87)	Qualitative studies (n = 6)
Global cognition and cognitive health constructs			
Global cognition^a^	47 (51)	46 (53)	1 (17)
Executive function	12 (13)	12 (14)	0
Processing speed	9 (10)	9 (10)	0
Visuospatial processing	4 (4)	4 (5)	0
Verbal fluency	7 (8)	7 (8)	0
Memory (large)	17 (18)	16 (18)	1 (17)
Verbal learning and memory	9 (10)	9 (10)	0
Working memory	5 (5)	5 (6)	0
Prospective memory	1 (1)	1 (1)	0
Verbal comprehension	1 (1)	1 (1)	0
Self-reported cognition, memory or cognitive decline	11 (12)	9 (10)	2 (33)
Mild cognitive impairment and dementia^b^			
Dementia (all types) diagnosis	38 (41)	35 (40)	3 (50)
Alzheimer disease	13 (14)	13 (15)	0
Vascular dementia	2 (2)	2 (2)	0
Self-reported dementia	5 (5)	5 (6)	0
Death associated with dementia	2 (2)	2 (2)	0
Mild cognitive impairment diagnosis	10 (11)	10 (11)	0
Self-reported healthy aging	1 (1)	0	1 (17)

^a ^ The global cognition category includes tests used to diagnose cognitive impairment. 

^b^ Categories are not mutually exclusive as each study could examine more than one association. The “dementia (all types)” category includes Alzheimer disease, self-reported dementia, vascular dementia and unspecified types of dementia. 

***Associations between SDoH and cognition***

We identified 410 main associations between SDoH and cognitive health constructs across the 93 individual studies included in this scoping review (see Supplemental Table 6 at https://osf.io/w4mqc). Of these 410 associations, 87 quantitative analyses reported 390 associations between SDoH categories and cognitive health constructs (see Supplemental Table 4 at https://osf.io/w4mqc) and six qualitative studies reported 20 descriptive thematic data linking SDoH and cognitive outcomes (see Supplemental Table 5 at https://osf.io/w4mqc).^20^-^25^ We also retrieved two trials that reported on interventions addressing SDoH (access to health care, rural or urban area of residence) and evaluated their impact on cognitive health.^26^,^27^

**Quantitative studies**

Of the 390 associations between SDoH and cognition and dementia in quantitative studies, education was the most frequently studied, followed by social support and household/individual income (see Table 4). 

**Table 4:** Frequencies of the main associations between SDoH and cognition reported in quantitative studies (n = 87)

SDoH	Cognitive health constructs, n								Dementia diagnosis, n				Cognitive impairment diagnosis, n	Death associated with dementia, n
	Global cognition^a^	Memory (all constructs)	Executive function	Verbal fluency	Processing speed	Visuospatial processing	Verbal comprehension	Attention	Dementia (all types)^b^	Alzheimer disease	Self-reported dementia	Vascular dementia		
Education	23	8	5	4	4	3	1	1	11	4	3	1	4	1
Social support	13	6	5	5	1	1	1	0	6	2	1	0	1	0
Income	11	3	3	2	0	1	0	0	8	0	0	0	1	1
Occupation or employment	11	3	2	1	2	0	0	0	3	1	1	1	0	2
Marital status	11	1	1	1	0	0	0	0	5	0	2	0	4	1
Environmental pollutants	1	1	1	0	1	1	0	0	9	5	0	0	0	1
Area of residence (rural/urban)	5	2	0	0	0	0	0	0	8	1	1	1	0	1
Living arrangement/ household composition	8	2	1	1	0	0	0	0	4	0	1	0	1	0
Ethnicity	7	3	1	1	0	0	0	0	2	1	0	0	2	0
Immigration status	6	1	0	1	0	0	0	0	3	1	0	0	1	1
Built environment	2	1	1	0	1	0	0	0	5	2	0	0	0	1
Neighbourhood deprivation or socioeconomic status	3	1	1	0	1	1	0	0	5	1	0	0	0	0
Access to health care	4	0	0	0	0	0	0	0	5	0	1	0	2	0
Housing	6	1	1	0	1	0	0	0	3	0	0	0	0	0
Primary language or language barriers	5	1	0	1	0	0	0	0	3	0	1	0	0	0
Loneliness	3	2	2	2	0	0	0	0	1	1	0	0	0	0
Occupational exposure	0	0	0	0	0	0	0	0	4	3	0	2	0	0
Childhood maltreatment	3	1	0	0	0	0	0	0	1	1	0	0	0	0
Sexual orientation	1	1	1	1	0	0	0	0	0	0	0	0	0	0
First Nations, Inuit or Mtis identity	2	0	0	0	0	0	0	0	1	0	0	0	0	0
Policy implementation	1	0	0	0	0	0	0	0	1	0	0	0	1	0
Gender identity or genderism	0	1	1	0	0	0	0	0	0	0	0	0	0	0
Food security	1	0	0	0	0	0	0	0	0	0	0	0	0	0
Knowledge of cognitive health	0	1	0	0	0	0	0	0	0	0	0	0	0	0
Marginalization	0	0	0	0	0	0	0	0	0	0	0	0	0	1
Religion	0	0	0	0	0	0	0	0	1	0	0	0	0	0
Stigma	1	0	0	0	0	0	0	0	0	0	0	0	0	0

**Abbreviation:** SDoH, social determinants of health. 

**Note:** This table shows the 390 associations between SDoH and cognitive health constructs described in the 87 retrieved quantitative studies. We identified all associations between each SDoH in each study and individual cognitive health constructs. Therefore, a specific SDoH reported in a study could have several associations with different cognitive health constructs. Each association is presented independently. 

^a^ The global cognition category includes tests used to diagnose cognitive impairment. 

^b^ Categories are not mutually exclusive as each study could examine more than one association. The “dementia (all types)” category includes Alzheimer disease, self-reported dementia, vascular dementia and unspecified types of dementia. 

**Qualitative studies**

The six qualitative studies were conducted in Alberta, Saskatchewan, Ontario and Newfoundland and Labrador with various populations, for example, people living with dementia, caregivers and health care workers, sex workers and Indigenous grandmothers.20-25 Access to health care was the most studied SDoH, followed by social support and area of residence (rural or urban). Macrosystemic SDoH such as stigma, religion, ethnicity, cultural background and maltreatment were also examined (see Table 5).

**Table 5:** Summary of qualitative studies reporting perceived changes in cognitive health constructs in response to SDoH

Study / Setting	Population	Social determinants of health	Selected quotes
Di Gregorio et al.(2015)^21^ Four communities in northern Ontario	2 people living with dementia, 15 care partners, 37 health care and social service providers, 17 other community members	Health care service providers‘ knowledge and awareness; access to health care; awareness and understanding of dementia; rural environment; community support networks; health and community care services	“Awareness is the first step in which one begins to have knowledge of the phenomenon: in this case, the signs and symptoms of dementia, the available resources, or people who have dementia.” “Despite lack of awareness of the signs and symptoms of dementia, people who were diagnosed with dementia were often more visible in rural environments.”
Pace (2020)^20^ NunatuKavut community, Newfoundland and Labrador	14 adults ≥ 50 years, family caregivers and home care workers	Social support	“Loneliness and isolation were described as risk factors for developing dementia, whereas social contact was seen as protective against cognitive decline. […] Participants described that seeing other people regularly was good for the brain and for mental wellness. Visiting and social gatherings were perceived to spur laughter, storytelling, and reminiscence, which were described as supportive of memory as well as the reinforcement of collective identity and shared experiences.”
Baumann et al. (2019)^22^ Urban settings in Toronto, Ontario	10 sex workers, women (n = 5) and transgendered women (n = 5), aged ≥18 years, in the Worksafe program	Sexual violence	“Some participants […] reported experiencing long-term consequences such as persistent memory loss, noise sensitivity and scars. Tiffany, who was strangled by an acquaintance, has persistent anxiety and memory loss, and shared that she has ‘short-term memory problems right now and [gets] constant headaches‘ […]”
Bacsu et al. (2020)^23^ Two rural communities in Saskatchewan	42 adults aged ≥ 60 years	Social support	“Rural older adults identified social engagement as being vital to supporting cognitive health. For instance, an older adult stated, ‘I feel a lot of brain health is due to mingling with others….‘ In particular, rural seniors described activities related to two subthemes: technology and social media; and community activities.”
Koehn et al. (2016)^24^ Chinese-Canadians in Greater Vancouver, British Columbia; anglophones in Calgary, Alberta; East Indian Canadians in Toronto, Ontario; francophones in Ottawa, Ontario	29 dementia and caregiver dyads	Access to health care; cultural background; education; immigration status; language barriers; quality of care; religion; social support; stigma	"Also important, however, is that credible sources be knowledgeable about the disease, which is where immigrant-serving agency staff typically fall short, since most lack the education in, and hence understanding of, the symptomology and trajectory of dementia and may not steer the person with dementia in the direction of appropriate services.”
Lanting et al. (2011)^25^ Northern communities in Saskatchewan	Three Indigenous grandmothers	Road access to northern communities (built environment); perceived cultural changes; culturally grounded health care	“[…] these data suggest common core cultural perceptions of aging and dementia identified by a group of [Indigenous] grandmothers. Additionally, perceived changes in culture are thought to underlie the increase in illness among [Indigenous] seniors and to negatively impact the process of aging.”

## Discussion

***Impact of SDoH on cognitive outcomes in Canada***

Using an ecological framework,^10^ we retrieved 159 publications covering 93 studies that encompassed numerous SDoH and cognitive health outcomes, including established associations (e.g. education, social support) and emerging or less-studied ones (e.g. working conditions, SDoH from the macrosystem). 

Our synthesis highlights researchers’ sustained efforts to understand the impact of SDoH on cognitive health in Canada. This scoping review complements existing international reviews^28^-^30^ and is the first to examine a comprehensive range of SDoH in relation to cognitive health in Canada. This synthesis provides information for policy makers and public health authorities on how Canadians’ living, work and aging conditions may affect cognitive outcomes.

***Geographical scope and representation of SDoH research***

About one-quarter (27%) of the included studies had nationwide coverage, while almost half were conducted in Ontario (27%) or Quebec (22%), the two most populous Canadian provinces. Nationwide studies, especially those with nationally representative samples, are useful for their generalizability and for identifying regional differences in the distribution of SDoH and health outcomes. However, we did not identify any studies conducted in Yukon, the Northwest Territories or Nunavut, and no province-specific findings from Nova Scotia or Prince Edward Island were available outside of multiprovince or nationwide studies. These gaps in the existing research highlight the need to research inadequately studied populations in Canada.

***Macrolevel and microlevel determinants and measurement challenges***

We found that 86% of the associations were with SDoH categorized in the microsystem, or the individual’s immediate environment, which aligns with the findings of a similar scoping review.^28^ Only one-third (33%) of the associations were with macrosystemic SDoH, which highlights a potential challenge in assessing these upstream, structural SDoH. 

The macrosystem, the outermost layer of the embedded systems surrounding the individual, has been described as the realm of the power systems driving SDoH and health disparities and as permeating throughout all other systems.^11^ Although macrosystemic SDoH have been less studied than those from the microsystem, we identified 31 studies assessing diverse macrosystemic SDoH, including knowledge of cognitive health, policy implementation, religion, stigma, sexual orientation and gender identity. 

The inclusion of qualitative studies enabled us to identify subjective experiences of SDoH, including stigma, discrimination or other associations with cultural background, ageism and genderism, which can be harder to assess otherwise. Future studies should focus on better ways to assess macrosystemic SDoH.

***Established and emerging determinants***

We found education and social support to be the most frequently studied SDoH, which aligns with the findings of other reviews.^28^-^30^ Social support, especially social engagement, social activities and the quality of relationships with caregivers, has been consistently studied in its relation to the cognitive health of individuals with or at risk for Alzheimer disease and related dementias.^30^ Marital status or loneliness may also play a role in cognitive decline, although the supporting evidence remains weaker than that for social engagement.^30^

Longitudinal studies have also observed education to be a protective factor against dementia-related pathologies, possibly due to greater cognitive reserve.^6^,^31^-^33^ The role of education in cognitive health is complex, as individuals with higher levels of education generally perform better on neurocognitive tests than those with a lower educational attainment.^34^ Thus, education could be considered either a key predictor or a confounding factor. However, education as a variable was usually self-reported formal education, either educational attainment or years of education, while cognitive stimulation from continuous learning throughout the lifespan remained largely unexamined in longitudinal studies.^35^

The role of occupational factors in cognitive health is increasingly recognized.^36^ Most of the studies in this review focused solely on occupation or employment status, but a few assessed exposure to chemicals or psychosocial hardships during work.^36^-^47^ Although many studies have examined exposure to work-related hazards (e.g. exposure to chemicals, work in the mining industry),^36^-^47^ the psychosocial aspects of work conditions (e.g. job strain, high psychological demand or low job control perception, job complexity) have only recently been investigated in a handful of studies, with mixed results.^36^,^42^-^45^

Quebec’s progressive occupational health and safety legislation provides a framework for protecting workers against physical and psychosocial hazards in the workplace, recognizing the broader health impacts that may extend into retirement.^36^,^48^ By requiring the assessment of workplace stressors such as job strain, high psychological demand, low job control and job complexity, and by mandating actions to mitigate them, this legislation has the potential to enhance cognitive health as well as cardiovascular and mental health outcomes.^49^,^50^ Quebec’s approach recognizes workplace psychosocial stressors as modifiable SDoH that can be addressed through targeted interventions and legislation.^48^,^51^

The built and natural environments also play a significant role in cognitive health; protective and risk factors depend on the availability of green spaces, walkable areas, social and physical activities, and other resources. Research conducted in Canada suggests that access to green spaces is associated with lower rates of dementia.^52^,^53^ Higher walkability scores are correlated with improved cognitive functioning in aging populations, underscoring the benefits of accessible and active living environments.^54^ Consistent with these findings, Canada’s national dementia strategy promotes community-based physical and social activities, particularly in rural and isolated regions, with built environments designed to support prevention.^4^-^6^ By developing accessible environments that encourage people to exercise, create art, play music and similar activities, built spaces can be leveraged to foster cognitive resilience.^55^,^56^ Such community-driven programs not only support dementia prevention but also highlight the potential of neighbourhood design as a modifiable SDoH with broad implications for physical, social and cognitive health.^55^,^56^

We retrieved only two interventional studies that targeted SDoH and examined their impact on cognitive health.^26^,^27^ This underscores the need for better integration of impact assessments in evaluations of public health initiatives on cognitive health in Canada. Future interventional studies could also examine the development of dementia-inclusive initiatives that address inequalities and inequities in SDoH, such as the age-friendly cities and communities proposed by the World Health Organization.^57^

***Methodological considerations: self-reported versus ecological data***

Most of the SDoH were assessed using self-reported measurements. Obtained through questionnaires or surveys, these are cost-effective and easy ways to assess a wide variety of SDoH from participants’ points of view.^58^ However, self-reports are subject to recall bias and social desirability bias, not least in the context of memory loss and cognitive decline.^58^ In addition, some microsystemic SDoH, such as social support and education, may be more likely to be studied because they are easily assessed through self-reports. 

Exosystemic SDoH, such as environmental and occupational exposures, were not as commonly evaluated. These SDoH were more often measured using ecological data such as dissemination areas or postal codes. Several barriers impede the use of ecological data, namely, data access, data linkage and regulatory processes.^59^ Future studies should further explore the potential of using ecological data to examine associations between possible risk factors and cognitive health outcomes.

***Strengths and limitations***

This scoping review has many strengths, including the exhaustive search strategy and inclusion of various SDoH associated with the full cognitive health spectrum. The focus on populations in Canada is a strength as the results can inform policy makers and health care professionals by providing directly relevant evidence. We have also highlighted opportunities for future research.

This review also has limitations. The focus on published Canadian literature restricted our ability to explore some SDoH because of a lack of external control groups. For example, we were unable to compare national health care systems, public programs or approaches. 

We limited the search to biomedical databases because of the large number of eligible studies retrieved, although a search of the grey literature might have identified other relevant publications or interventions and data on other or under-represented populations. In addition, data extraction was conducted by only one reviewer. 

We could not combine the information on study designs using the study as the unit of analysis because the various publications describing the same study may have used different study designs. Finally, the various study designs and analytical approaches did not allow for meta-analyses of the associations. Our results may, however, guide the development of future systematic reviews to explore these associations.

## Conclusion

This scoping review identified 159 publications covering 93 studies describing associations between SDoH in 29 categories and cognitive health outcomes on the continuum from healthy cognition to dementia. Along with showing diverse associations, we also highlighted the complexity of evaluating social determinants and the value of using a broad range of data sources and data collection approaches within studies for a better coverage of the SDoH. Our findings show the importance of examining a wide range of SDoH, better integrating the chains of influence between SDoH, and designing both targeted and global interventions that would act efficiently upon cognitive health. Future research should also focus on integrating more measures of positive cognitive function and healthy aging to cover this continuum of cognitive health.

## Funding

We thank the Centre for Surveillance and Applied Research and the Centre for Health Promotion from the Health Promotion and Chronic Disease Prevention Branch of the Public Health Agency of Canada for their financial support.

## Acknowledgements

We thank the Centre for Surveillance and Applied Research and the Centre for Health Promotion in the Health Promotion and Chronic Disease Prevention Branch of the Public Health Agency of Canada for their valuable expertise and feedback; the Public Health Agency of Canada Library for their help in validating the search strategy; and Dominique Parisien from the Public Health Capacity and Knowledge Mobilization Unit, the Regulatory, Operation and Emergency Management Branch of the Public Health Agency of Canada, for her help in the realization of this project and relevant knowledge transfer. 

## Conflicts of interest

The authors have no conflicts of interest.

## Authors’ contributions and statement

SO: Conceptualization, data curation, formal analysis, investigation, methodology, writing—original draft, writing—review and editing.

TR: Conceptualization, data curation, formal analysis, investigation, writing—original draft, writing—review and editing.

MLR: Conceptualization, project administration, supervision, validation, writing—review and editing.

LL: Conceptualization, project administration, supervision, validation, writing—review and editing.

The content and views expressed in this article are those of the authors and do not necessarily reflect those of the Government of Canada.
